# Statins and/or fibrates for diabetic retinopathy: a systematic review and meta-analysis

**DOI:** 10.1186/s13098-019-0488-9

**Published:** 2019-11-08

**Authors:** Vânia Mozetic, Rafael Leite Pacheco, Carolina de Oliveira Cruz Latorraca, Rachel Riera

**Affiliations:** 1 Discipline of Evidence-Based Medicine, Escola Paulista de Medicina (EPM), Universidade Federal de São Paulo (Unifesp), São Paulo, Brazil; 2 Instituto Dante Pazzanese de Cardiologia, São Paulo, Brazil; 3 Centro de Pesquisa Médica, Centro Universitário São Camilo, São Paulo, Brazil; 4 Center of Health Technology Assessment, Hospital Sirio-Libanês, São Paulo, Brazil

**Keywords:** Diabetic retinopathy, Statin, Fibrates, Evidence-based practice, Evidence-based medicine, Systematic review

## Abstract

Evidence from observational studies have found a relationship between serum cholesterol and diabetic retinopathy (DR). Apart of the assumption that cholesterolemic control has benefits for patients with diabetes with or without retinopathy, the effects of lipid-lowering drugs have not been properly mapped and critically assessed so far. The objective of this study was to evaluate the effects of statins and/or fibrates on prevention and progression of DR. We conducted a Systematic review of randomized controlled trials (RCTs) following the Cochrane Handbook for Systematic Reviews of Interventions and reported in accordance to PRISMA Statement. GRADE approach was used to summarize the certainty of the evidence. Eight RCTs that fulfilled our eligibility criteria were included, assessing the effects of fibrates (n = 4), statins (n = 3) and fibrate plus statins (n = 1) for therapy (n = 8) or prevention (n = 4) of DR. Overall, the main concern regarding risk of bias assessment was due to incomplete outcome data because high rate of losses in five RCTs. Furthermore, the risk of reporting bias was rated unclear due the lack of previously published protocol in seven RCTs. Fibrates seemed to be associated with a 45% risk reduction of macular edema incidence (Relative Risk 0.55, 95% confidence interval of 0.38 to 0.81, 1309 participants, 2 RCTs, I^2^ = 0%, low certainty of the evidence). The certainty of evidence for other outcomes was also very low or low, and we are uncertain regarding the effects of fibrates for DR. Overall, adverse events seemed to be similar between fibrate and placebo, but again based on the width of the confidence intervals, an important increase of adverse events cannot be rule out. The combination statin/fibrate did not seem to have benefit for visual acuity but is likely that further studies can modify this estimate since the current evidence is limited. Adverse events and quality of life were not measured or reported. Concluding, this study found eight RCTs, with limited methodological quality, that assessed the effects of fibrates and/or statins for DR. Based on these findings, we are uncertain about the effects of statins for DR. Fibrates seemed to reduce the incidence of macular edema (low certainty evidence) without increase adverse events (low to very low certainty evidence).

*Number of Protocol registration* PROSPERO CRD42016029746.

## Background

In 2010 worldwide, approximately 833,690 people presented blind due to diabetic retinopathy (DR) induced blindness and 3.7 million were visually impaired. Along 10 years (from 1990 to 2010), DR-induced blindness increased by around 27% and DR-related visual impairment by 64% [1]. These numbers make DR a growing public health problem, with an important burden on health status and economic systems [2].

The high blood glucose is the trigger to unleash a series of neurological and vascular changes that culminate in loss of vision. Glycemic control persists as the best way to postpone the onset and delay the progression of DR, but it does not seem to be enough [3–5].

The main line of treatment for DR includes laser photocoagulation [6, 7], anti-VEGF (vascular endothelial growth factor) [8–10] and corticosteroids [11]. Lipid-lowering drugs have been proposed and used in clinical practice [12]. The rationale is that cholesterolemic control may have effects on delaying the progression of DR. The evidences from observational studies are inconsistent, and some studies have found a close relationship between serum cholesterol and DR development [13, 14].

Although the pathophysiology of DR is coherent with the cholesterolemic control, the assessment of the effects of lipid-lowering drugs has not been properly mapped in the literature. Thus, the objective of this systematic review is to synthetize all RCTs that assessed the benefits and harms of the lipid-lowering drugs (statin and/or fibrates) for the prevention and treatment of DR.

## Methods

### Study design and setting

We performed a systematic review according to the Cochrane Handbook for Systematic reviews of interventions [15]. The manuscript was prepared in accordance to the recommendations of *Preferred Reporting Itens for Systematic Reviews and Meta*-*analysis* (PRISMA) [16]. The protocol was published prospectively [17] and registered in PROSPERO database **(**http://www.crd.york.ac.uk/PROSPERO/**)** under the number CRD42016029746. This study was conducted at the Evidence-based Healthcare Post-graduation Program of Universidade Federal de São Paulo.

### Inclusion criteria

#### Types of studies

We included only parallel randomized clinical trials (RCTs), as they are the best study design to assess the effects of an intervention.

#### Types of participants

We intended to include all patients (regarding age or sex) with type 1 or 2 diabetes, with or without nonproliferative retinopathy for treatment and prevention, respectively. We excluded studies that evaluated patients with proliferative retinopathy. If one study presented mixed data for patients with non proliferative and proliferative, we contacted the authors to further information.

#### Types of interventions

We considered all RCTs assessing statin or fibrate, compared to placebo, no intervention, or a different type of statin or fibrate. We only considered combined therapy between these two drugs if the effects of one intervention could be assessed in isolation. We considered RCTs with any dose, duration course of the intervention.

#### Outcomes

We focused in clinical relevant outcomes that could directly affect patients and health care system. We included studies which considered at least one of the following:

#### Primary outcomes

1. Incidence of DR: proportion of participants with DR incident, as defined and measured by primary author of primary study, including the definition of non‐proliferative DR (Early Treatment Diabetic Retinopathy Study—ETDRS-final score of 35 or greater, by stereoscopic color fundus photographs of eye) [18] or incidence of macular edema.
2. Progression of DR: proportion of participants with progression of DR, as defined and measured by primary author of primary study, as example (but not restricted to): two‐step or greater progression from baseline on the ETDRS final scale based on evaluation of stereoscopic color fundus photographs or progression of macular edema.
3. Serious adverse events: proportion of participants with at least one serious adverse event (i.e., those that are immediately life-threatening, or resulted in hospitalization, incapacity, malignant disease, or death).

#### Secondary outcomes

4. Visual acuity: proportion of participants with decrease of visual acuity (any decrease) measured by Snellen or LogMAR charts [19, 20];
5. Progression to proliferative DR: proportion of participants that developed proliferative DR, as defined and measured by primary author of primary study, including the need of laser photocoagulation.
6. Quality of life: measured by a validated vision-related scale.
7. Any adverse event: proportion of participants with at least one adverse event.
  We consider the outcomes at short-term (less than 6 months) and long-term (6 months or more).

### Searching for studies

#### Electronic Search

We performed systematic and sensitivity searches of the literature at the following electronic databases:

- Cochrane Central Register of Controlled Trials (CENTRAL, via Wiley);
- MEDLINE (via Pubmed);
- EMBASE (via Elsevier);
- Literatura Latino Americana em Ciências da Saúde e do Caribe (LILACS, via Biblioteca Virtual em Saúde-BVS);
- ClinicalTrials.gov (http://www.clinicaltrials.gov);
- World Health Organization (WHO) International Clinical Trials Registry Platform (ICTRP, apps.who.int/trialsearch/).
- OpenGrey (http://www.opengrey.eu).

We did not impose language, data or status from the publication limitations. The full search strategy for each database is presented in Additional file 1.

#### Hand search

We also assessed reference lists of all included studies and review articles for additional references. We asked for specialists in the field to inquire regarding ongoing studies.

### Selection of studies

The screening process was performed in two stages. In the first stage, two authors (VM and RLP) independently screened the references retrieved by the search strategy and selected the abstracts of potential eligible SRs. The selected abstracts were then read in full text (second stage) by two independents authors (VM and RLP) to check if they indeed fulfilled the inclusion criteria. Any disagreements in the screening process was solved by consulting a third researcher (RR). This process was performed using the Rayyan software [21].

### Data extraction

Two authors (VM and RLP) extracted the relevant data regarding characteristics, methodology and outcomes through a data collection form. Any disagreement in this stage was also solved by a third researcher (RR).

### Risk of bias assessment

Two authors (RLP and VM) assessed the risk of bias from all included studies using the Risk of Bias table from Cochrane Library. The risk of bias of each study was assessed in seven domains: random sequence generation, allocation concealment, blinding of participants and personnel, blinding of outcomes assessors, incomplete outcome data, selective outcome reporting and other potential threats.

Each domain was judge as having low risk of bias (if the domain was adequate), high risk of bias (if the domain was inadequate) or unclear risk of bias (if there was no enough information to support the judgment). All of the judgements were performed by following the recommendations from Chapter 8 of the Cochrane Handbook [15]. The reasons for each judgment were presented in this manuscript. A third researcher (RR) was consulted in any disagreement in the risk of bias assessment.

### Measures of treatment effect

We estimated the treatment effect for all outcomes as risk ratios (with 95% confidence interval).

### Unit of analysis issues

We did not impose restriction regarding the unit of analysis. We included any used by the primary authors (the patient, the worst eye and each eye). We only pooled together studies that used the same unit of analysis.

### Missing data

We contacted the authors by email for inquiring any missing data that we considered relevant (e.g. result data or methodological aspects) and that would contribute to the analysis.

### Heterogeneity assessment

We assessed the clinical, methodological and statistical heterogeneity from all included studies. Clinical heterogeneity was assessed regarding clinical characteristics from the populations, concurrent or prior treatments, comorbidities. Methodological heterogeneity was assessed regarding risk of bias and performance of the included studies. The assessment of statistical heterogeneity was performed using the Chi square test (with a significance margin corresponding to a *p* value of 0.1 or less) and the I^2^ statistics (values higher than 50% were considered to having substantial inconsistency). We also intended to investigate any reasons for heterogeneity by performing subgroup or sensitivity analysis.

### Publication bias assessment

We planned to perform an assessment of the publication bias by visual inspection of funnel plots. This was not possible because we did not perform any meta-analysis with 10 or more pooled studies.

### Data synthesis

We pooled results (on dependence of data availability and homogeneity) by performing a random-effects model meta-analysis using the Review Manager 5.3 software [22]. We also presented the results narratively when meta-analysis was not possible.

### Sensitivity and subgroup analysis

Subgroup analyses for the primary outcomes would be conducted considering the following groups: diabetic macular edema status of the patient. Sensitivity analysis would be conducted to assess the impact of exclusion of studies with high risk of bias (those judged to have *high risk* of bias in at least one of the domains: generation of randomization sequence, allocation concealment, and blinding).

### Assessing the certainty of the body of the evidence

We assessed the certainty of the body of the evidence by using the Grading of Recommendations Assessment, Development and Evaluation (GRADE) criteria (risk of bias, imprecision, inconsistency, indirectness and publication bias) [23]. We followed the recommendations of the chapter 11 from the Cochrane Handbook to perform the assessment of all primary outcomes [15]. All decisions to downgrade or upgrade the evidence were presented in this report. We created summary of findings tables for the comparisons statins versus placebo, fibrates versus placebo and fibrate plus statin versus statin alone using the GRADEpro software [24].

## Results

### Search results

**Fig. 1:**
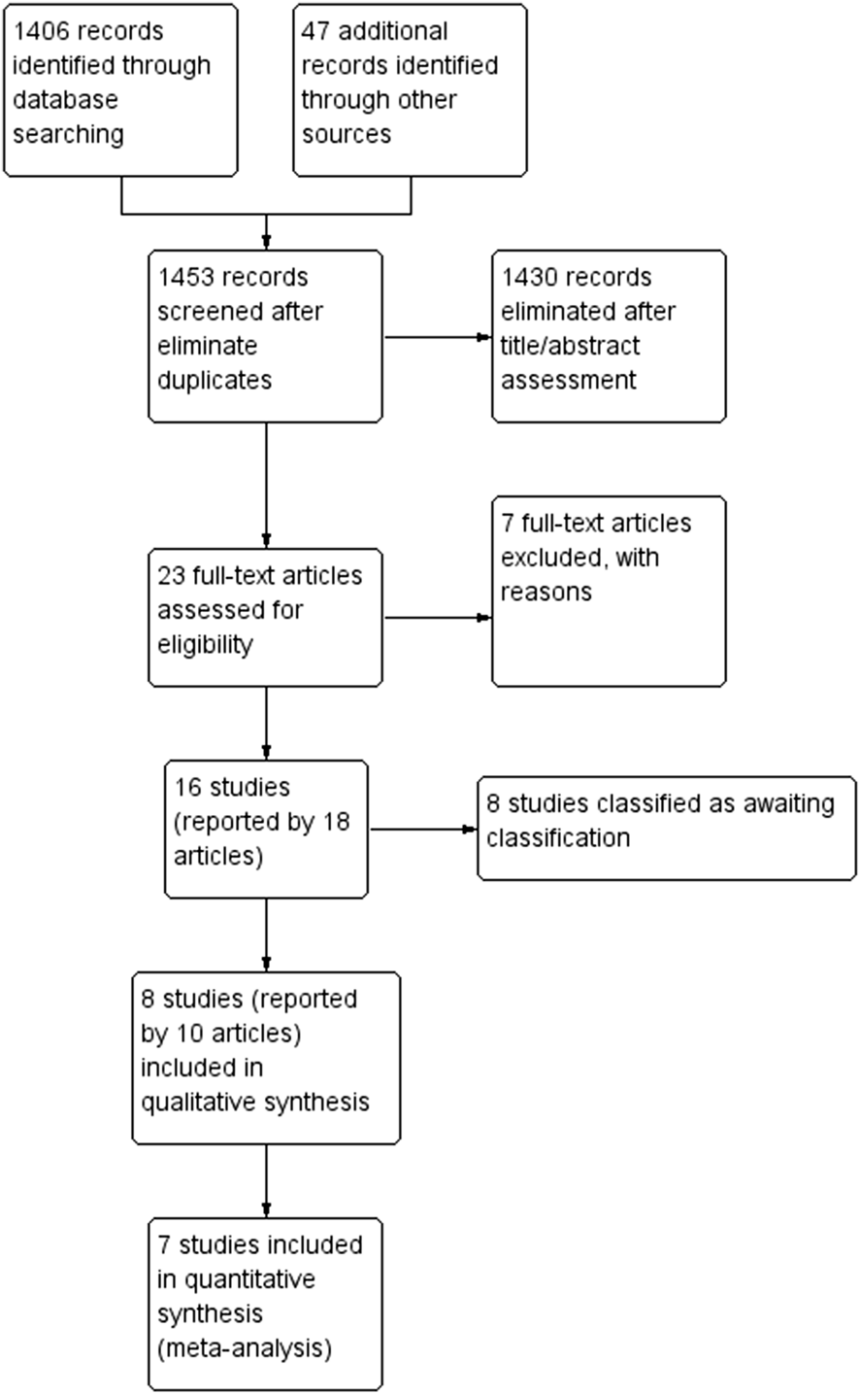
PRISMA flow diagram for the screening process of studies

The search was conducted at February 1st, 2018. Our initial search retrieved 1453 records and after reading titles and abstracts, 1430 records were eliminated. From 23 potentially eligible studies, seven were excluded with reasons (Additional file 2) [25–31] and eight were remained awaiting classification (Additional file 3) [32–39]. Finally, eight studies fulfilled our eligibility criteria [40–47]. The PRISMA flow diagram for the screening process in presented in Fig. 1.

### Characteristics of included studies

We included eight RCTs assessing the effects of fibrates (n = 4), statins (n = 3) and fibrate plus statins (n = 1) for therapeutic (n = 8) or prevention (n = 1) of DR. The main characteristics of these RCTs are presented in Table 1.

**Table 1:** Main characteristics of included studies

Study (author, year)	Population	Interventions	Outcomes of interest for this review	Time point for outcome measurement (months)	Funding/register
Chew, 2014 (ACCORD Eye)	Type 2 diabetes, moderate dislipidemia, established cardiovascular disease or cardiovascular risk factors (n = 1594)	Group 1: fenofibrate 160 mg/day *plus* simvastatin (n = 806) Group 2: placebo *plus* simvastatin (n = 787)	Incidence of DR (ETDRS) Progression of DR (ETDRS) Progression for proliferative disease (participant referred to photocoagulation). Visual acuity (Logarithm of the Minimum Angle of Resolution, LogMAR)	48	National Heart, Lung, and Blood Institute, National Institutes of Health (NHI), National Institute of Diabetes and Digestive and Kidney Diseases, the National Eye Institute, the national Institute on Aging, Center for Disease Control and Prevention Tablets of fenofibrate, equipments and supplies were provided by a pool of pharmaceutics companies
Cullen, 1974	Non-proliferative diabetic retinopathy (n = 40)	Group 1: clofibrate 2 g/day (n = 20) Group 2: placebo (n = 20)	Progression of DR (hard exudates progression, similar to ETDRS). Progression for proliferative disease (participant referred to photocoagulation). Visual acuity (Snellen). Mortality	24	Tablets of clofibrate and placebo were supplied by Imperial Chemical Industries Ltda Ross Foundation, Scotland
Emmerich, 2009	Non-proliferative diabetic retinopathy (n = 296)	Group 1: etofibrate 1 g/day (n = 148) Group 2: placebo (n = 148)	Incidence of DR (macular edema) Progression of DR Visual acuity Adverse events (counting of severe and mild events and rate of participants with any adverse events) Mortality	6 and 12	None
Gupta, 2004	Non-proliferative diabetic retinopathy with clinically significant macular edema (n = 30)	Group 1: atorvastatin 10 mg/day (n = 15) Group 2: no intervention (n = 15) *Both groups received also Nd Yag Green laser (532* *Nm)*	Progression of DR (macular edema, distribution of hard exudates) Visual acuity	1, 5; 3 and 4, 5	Not described
Keech, 2007 (FIELD Sudy)	Non-proliferative diabetic retinopathy with no clinically significant macular edema; no diabetic retinopathy (n = 1012)	Group 1: micronised fenofibrate 200 mg/day (n = 512) Group 2: placebo (n = 500)	Incidence of DR (macular edema) Progression of DR (ETDRS and hard exudates) Progression for proliferative disease (ETDRS) Visual acuity (Snellen) Mortality	42 and 60	Laboratories Fournier SCA
Massim, 2014 (MacuFen Study)	Non-proliferative diabetic retinopathy with macular edema (n = 110)	Group 1: fenofibrate 135 mg/day (n = 57) Group 2: placebo (n = 53).	Progression of DR (ETDRS, edema macular and exudate) Progression for proliferative disease (laser need) Visual acuity (Snellen). Severe adverse events	12	Laboratories Fournier SCA (previously Abbott)
Narang, 2012	Non-proliferative diabetic retinopathy with clinically significant macular edema (n = 30)	Group 1: atorvastatin 20 mg/day (n = 15) Group 2: placebo (n = 15). *Both groups received also Nd Yag Green laser (532* *Nm)*	Progression of DR (distribution of hard exudates) Visual acuity (Snellen)	6	None
Sen, 2002	Non-proliferative diabetic retinopathy with no clinically significant macular edema (n = 50)	Group 1: simvastatin 20 mg/day (n = 25). Group 2: placebo (n = 25)	Incidence of DR (macular edema) Progression of DR (fundus eye photography) Visual acuity (Snellen)	3 and 6	Ranbaxy Laboratories

*DR* diabetic retinopathy, *ETDRS* Early Treatment Diabetic Retinopathy Research Group

### Methodological quality and risk of bias of included RCTs

**Fig. 2:**
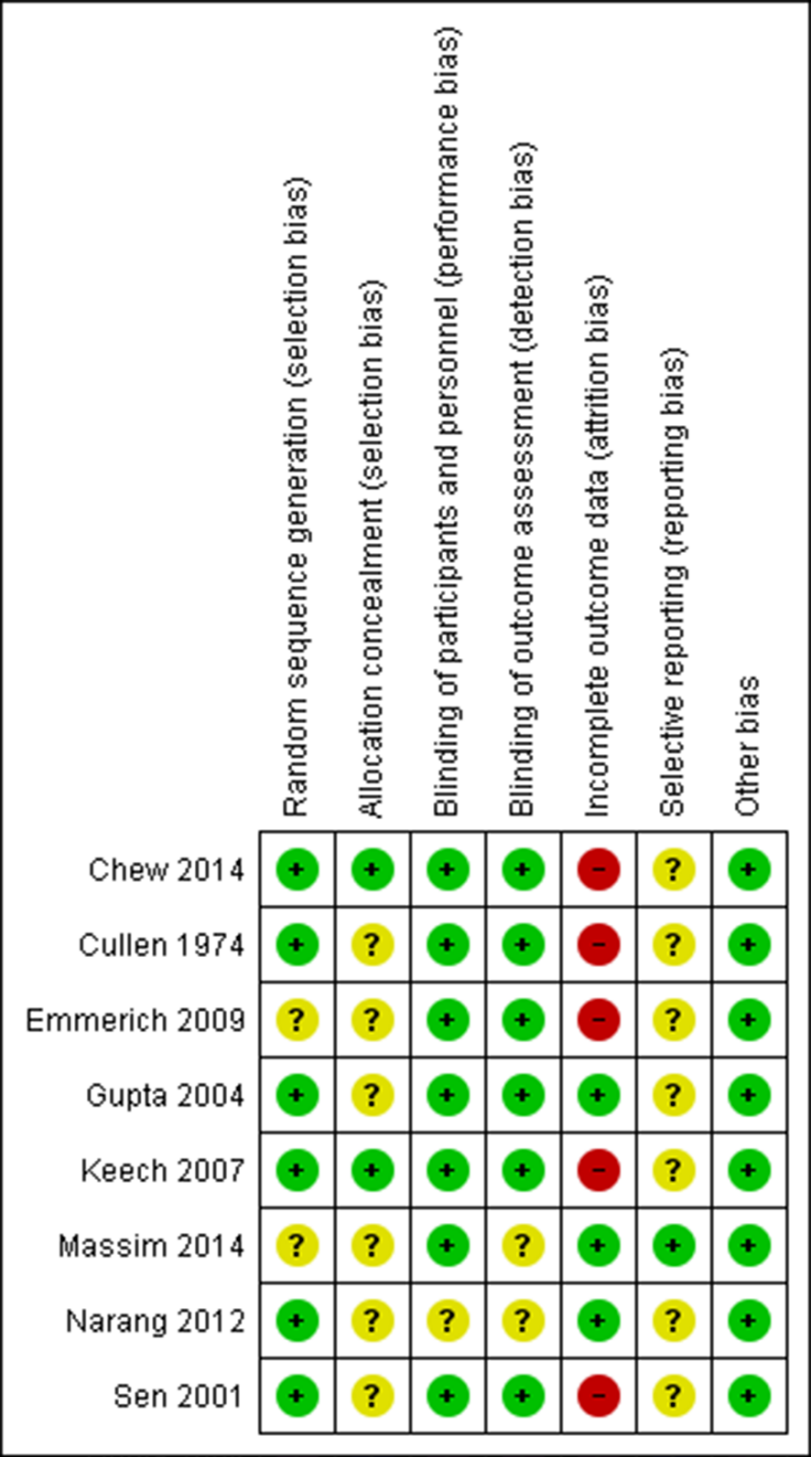
Risk of bias summary

The results of the risk of bias judgment and the reason for each judgment are presented in Additional file 4. A summary of the risk of bias is presented in Fig. 2. The critical point regarding risk of bias are related to incomplete outcome, judged as high for five RCTs [40–42, 44, 47] and low for three of them [43, 45, 46]. All RCTs, except Massin 2014, presented unclear risk of selective reporting due to absence of a previously available protocol or a retrospectively registered protocol.

### Effects of intervention and certainty of the body of the evidence

The eight included studies assessed three comparisons: (1) statin versus placebo [43, 46, 47], (2) fibrate versus placebo [41, 42, 44, 45] and statin plus fibrate versus statin [40]. The certainty of the evidence assessed by GRADE approach is presented in Additional files 5, 6, 7.

### Statin versus placebo

#### Incidence of DR (proportion of patients that developed DR)

One RCT assessed this outcome by the incidence of macular edema [47]. The risk of macular edema was 0/25 in simvastatin group and 4/25 in placebo group (p = 0.009).

#### Progression of DR (proportion of participants with retinopathy progression)

**Fig. 3:**
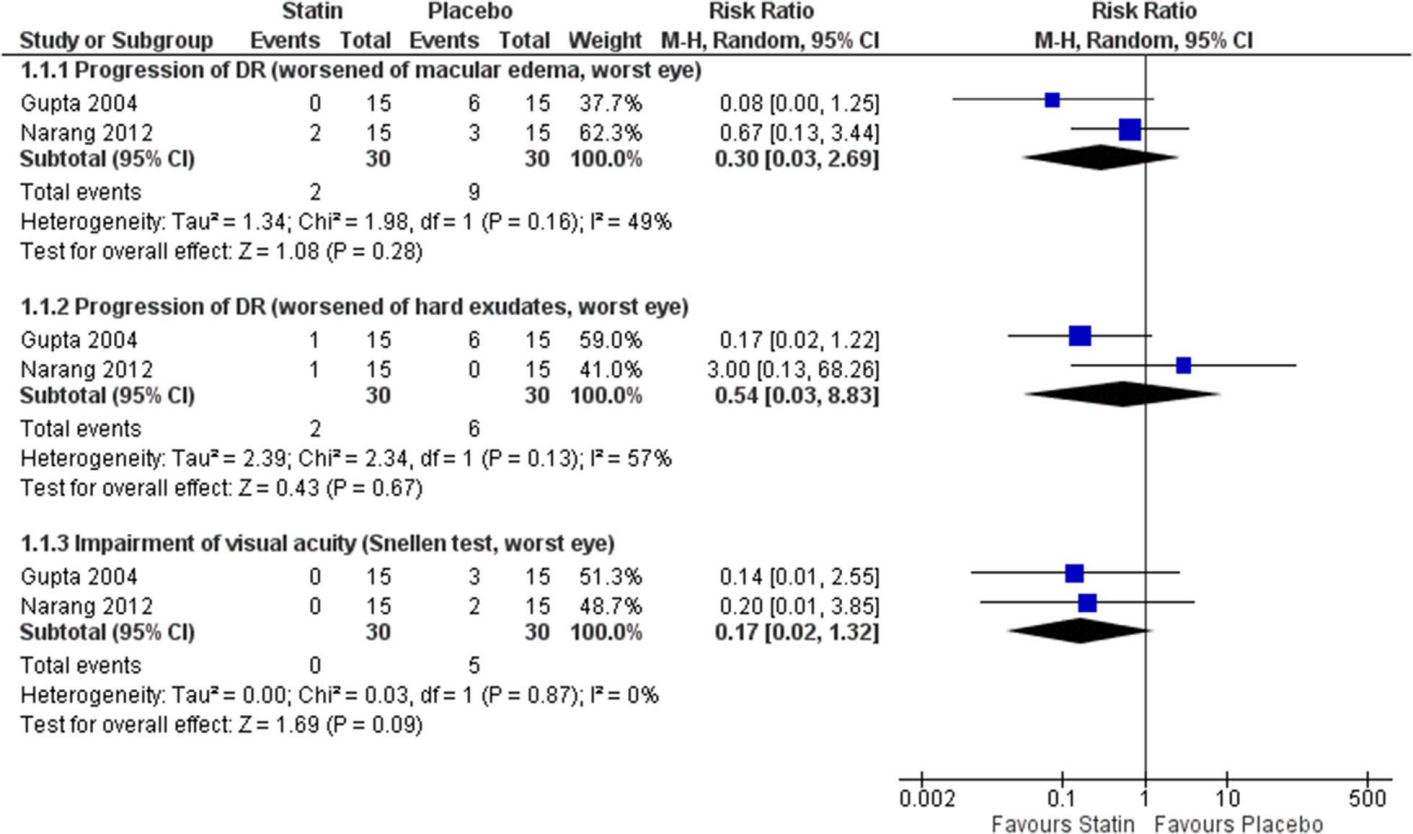
Comparison: statin versus placebo. Outcomes: progression of DR (impairment of macular edema and hard exudates) and visual acuity (impairment)

Three RCTs assessed this outcome, considering the progression of macular edema [43, 46], progression of hard exudates [43, 46] and fundus eye photography [47]. The unit of analysis also differed among the studies: worst eye [43, 46] and the individual [47]. In Sen 2002, the risk of progression of DR (fundus eye photography) was 0/25 in simvastatin group and 7/25 in placebo group (reported as non significant, p value not provided). The meta-analysis showed no difference between statin and placebo for macular edema, but this analysis was very imprecise and the confidence intervals includes an important reduction or increase in the risk (RR 0.30, 95% CI 0.03 to 2.69; participants = 60; studies = 2; I^2^ = 49%; very low certainty of the evidence) neither for hard exudates, but the imprecision was also important, and the direction of the effect is also uncertain (RR 0.54, 95% CI 0.03 to 8.83; participants = 60; studies = 2; I^2^ = 57%; very low certainty of the evidence) (Fig. 3).

Visual acuity (proportion of participants with 2 or more lines decrease of visual acuity). Three RCTs assessed this outcome, two studies considered the worst eye as unity of analysis [43, 46] and the meta-analysis was also very imprecise, with the data being compatible with an important increase or decrease in the relative risk (RR 0.17, 95% CI 0.02 to 1.32; participants = 60; studies = 2; I^2^ = 0%; very low certainty of the evidence). One study considering the individual as unity of analysis and found a worsening of visual acuity of 0/25 participants in simvastatin group and 7/25 participants in placebo group (p = 0.009) [47] (Fig. 3).

None of included RCTs assessed adverse events, quality of life and progression to proliferative DR.

### Fibrates versus placebo

#### Incidence of DR (proportion of patients that developed DR)

**Fig. 4:**
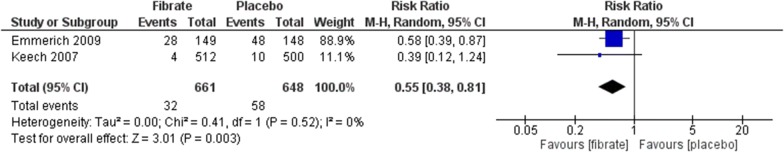
Comparison: fibrate versus placebo. Outcomes: incidence of DR (macular edema), long-term

Two RCTs assessed this outcome at long-term and considered the incidence of macular edema [42, 44]. The meta-analysis found benefit with fibrate use (RR 0.55, 95% CI 0.38 to 0.81; participants = 1309; studies = 2; I^2^ = 0%; low certainty of the evidence) (Fig. 4). The certainty of evidence was consider low due imprecision and risk of bias, which means that future studies may change the estimative around the incidence of macular edema in patients using fibrates.

#### Progression of DR

**Fig. 5:**
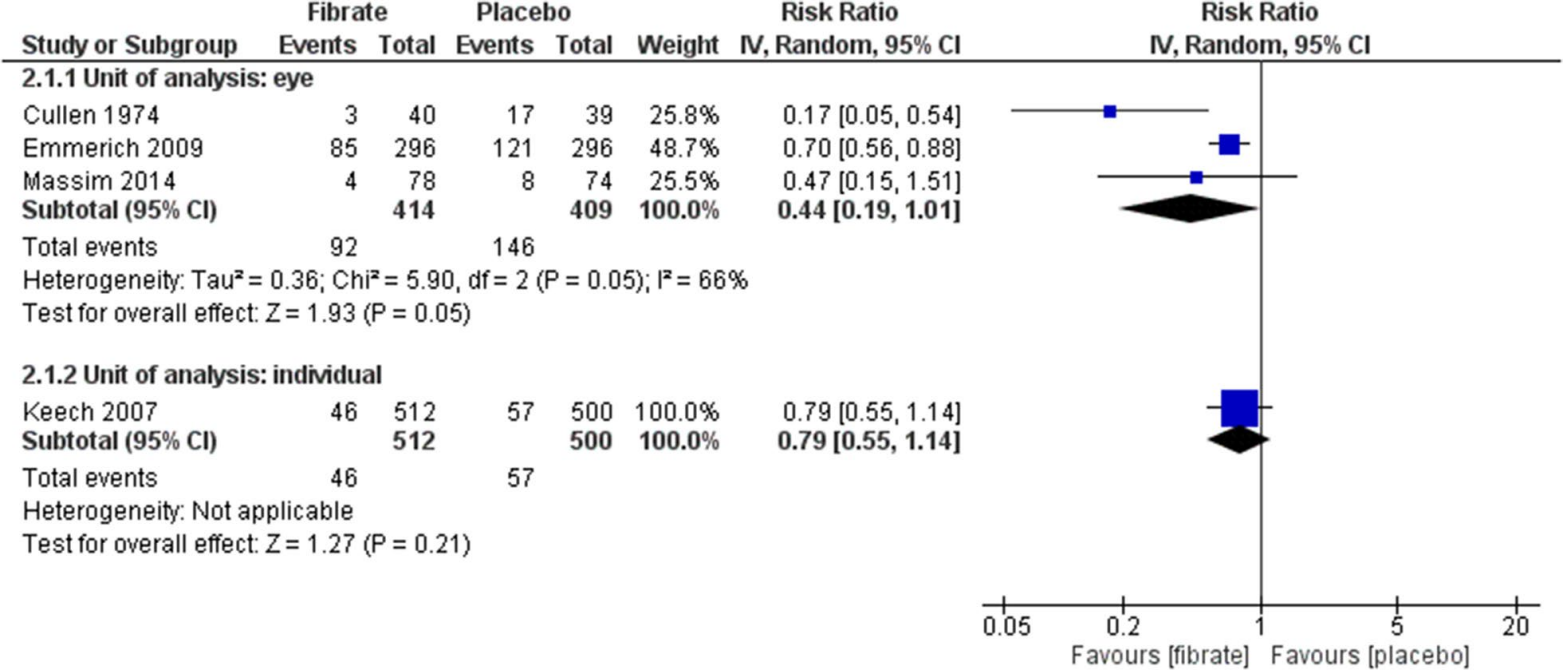
Comparison: fibrate versus placebo. Outcomes: progression of DR, long-term (ETDRS or similar)

**Fig. 6:**
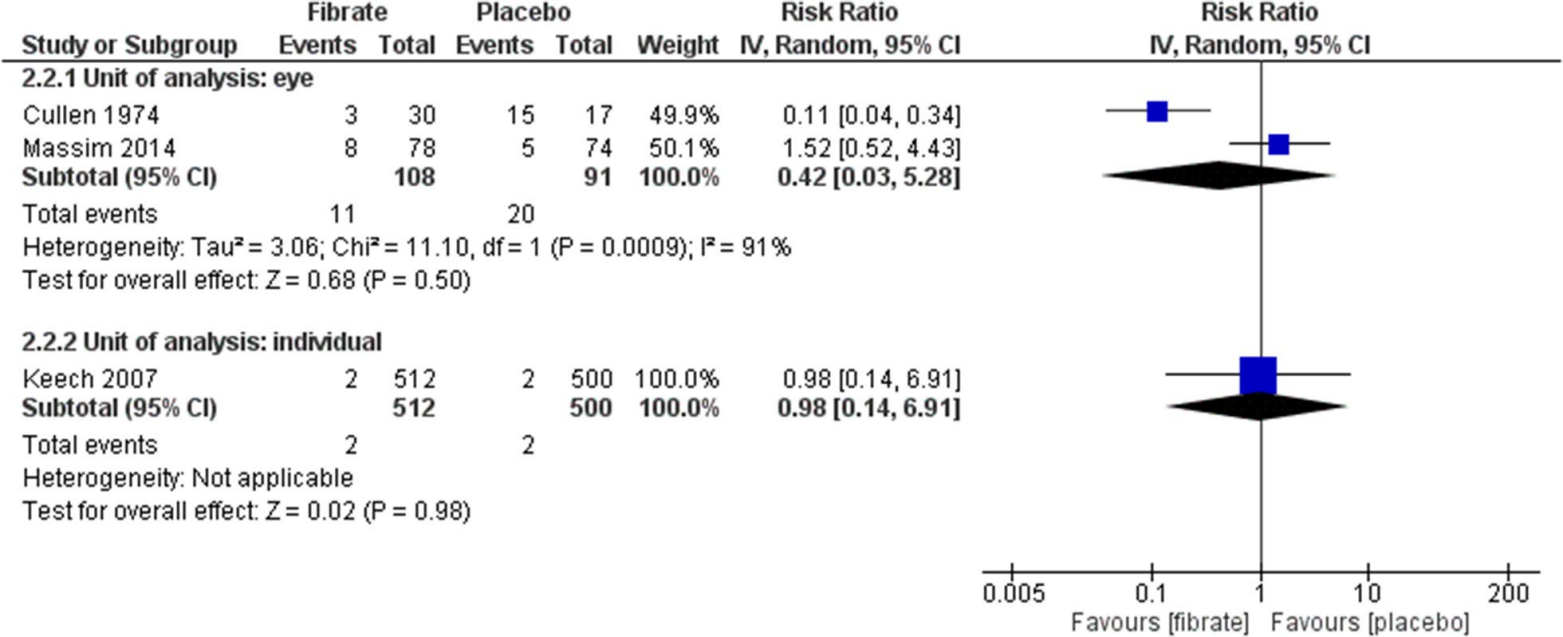
Comparison: fibrate versus placebo. Outcomes: progression of DR, long-term (hard exudates)

Four RCTs assessed this outcome at long-term using EDTRS or similar [41, 42, 44, 45]. The meta-analysis found no benefit with fibrate neither considering the eye as unity of analysis (RR 0.44, 95% CI 0.19 to 1.01; participants = 823; studies = 3; I^2^ = 66%; very low certainty of the evidence) nor the individual (RR 0.79, 95% CI 0.55 to 1.14; participants = 1012; studies = 1; certainty of the evidence not assessed) (Fig. 5). The same was observed using hard exudates for eye (RR 0.42, 95% CI 0.03 to 5.28; participants = 199; studies = 2; I^2^ = 91%; very low certainty of the evidence) and the individual as unity of analysis (RR 0.98, 95% CI 0.14 to 6.91; participants = 1012; studies = 1; certainty of the evidence not assessed) (Fig. 6). All of these estimates were considered at very low certainty of evidence or had a large confidence of interval, meaning that the effects of the interventions may be substantial different than the point estimates.

Visual acuity (proportion of participants with 2 or more lines decrease of visual acuity). Four RCTs assessed this outcome [41, 42, 44, 45] but no meta-analysis was conducted to due clinical heterogeneity among the studies, different unity of analysis and lack of numeric data. Cullen reported no statistical difference between groups considering the number of eyes with worsened visual acuity (8/40 for fibrate versus 9/39 for placebo, p-value was not provide; certainty of the evidence not assessed) [41]. Keech 2007 reported no difference between groups for the number of participants with worsened visual acuity (97/512 (29.1%) for fibrate *versus* 90/500 (30.7%) for placebo, p = 0.67; low certainty of the evidence) [44]. Two RCTs poorly reported data for this outcome and stated that no significant difference was found [42, 45].

#### Progression to proliferative DR

**Fig. 7:**
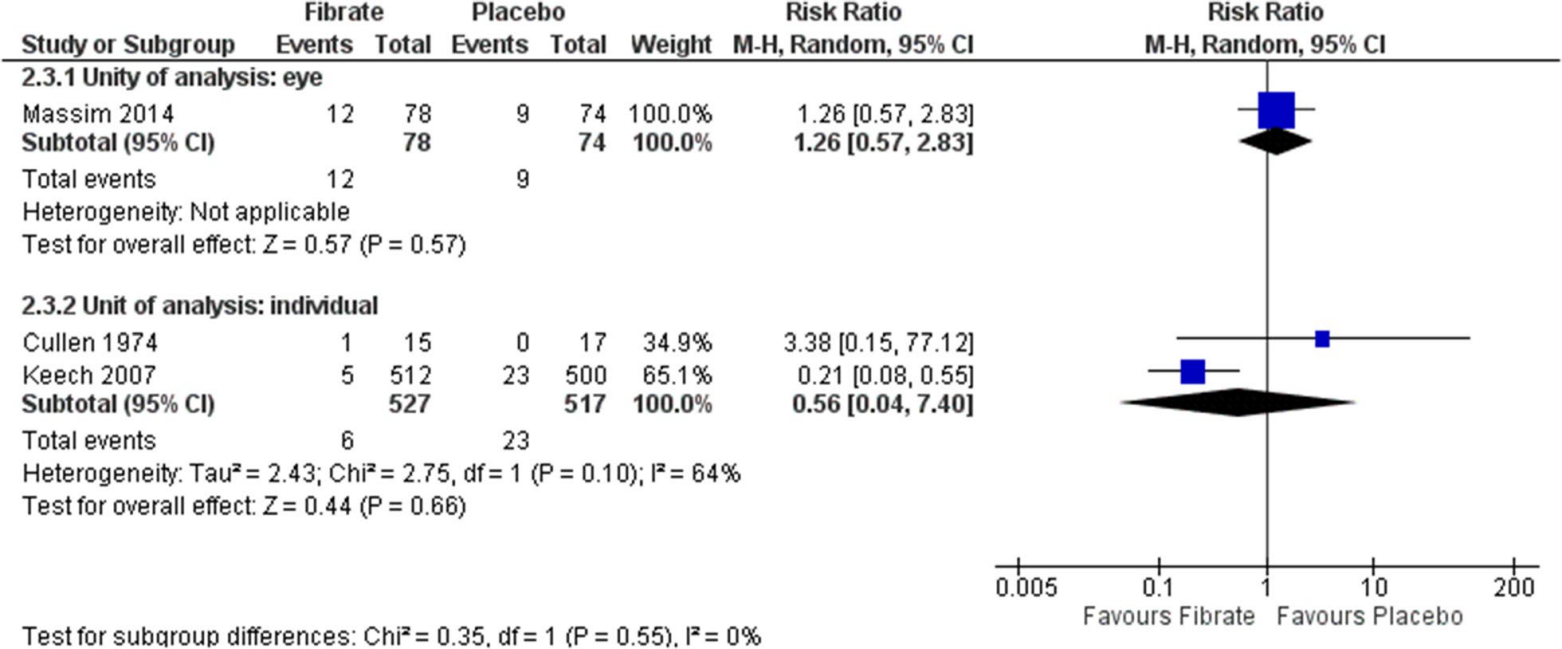
Comparison: fibrate versus placebo. Outcomes: progression to proliferative DR

Three RCTs assessed this outcome by presence of neovascularization or necessity of photocoagulation [41, 44, 45]. No difference was found neither considering eye as unity of analysis (RR 1.26, 95% CI 0.57 to 2.83; participants = 152; studies = 1; certainty of the evidence not assessed) nor the individual (RR 0.56, 95% CI 0.04 to 7.40; participants = 1044; studies = 2; I^2^ = 64%; very low certainty of the evidence) (Fig. 7). The low number of events also reduce the precision of the estimates, and the confidence intervals were broad and includes both important reduction or increase in the risk with the intervention.

#### Adverse events

**Fig. 8:**
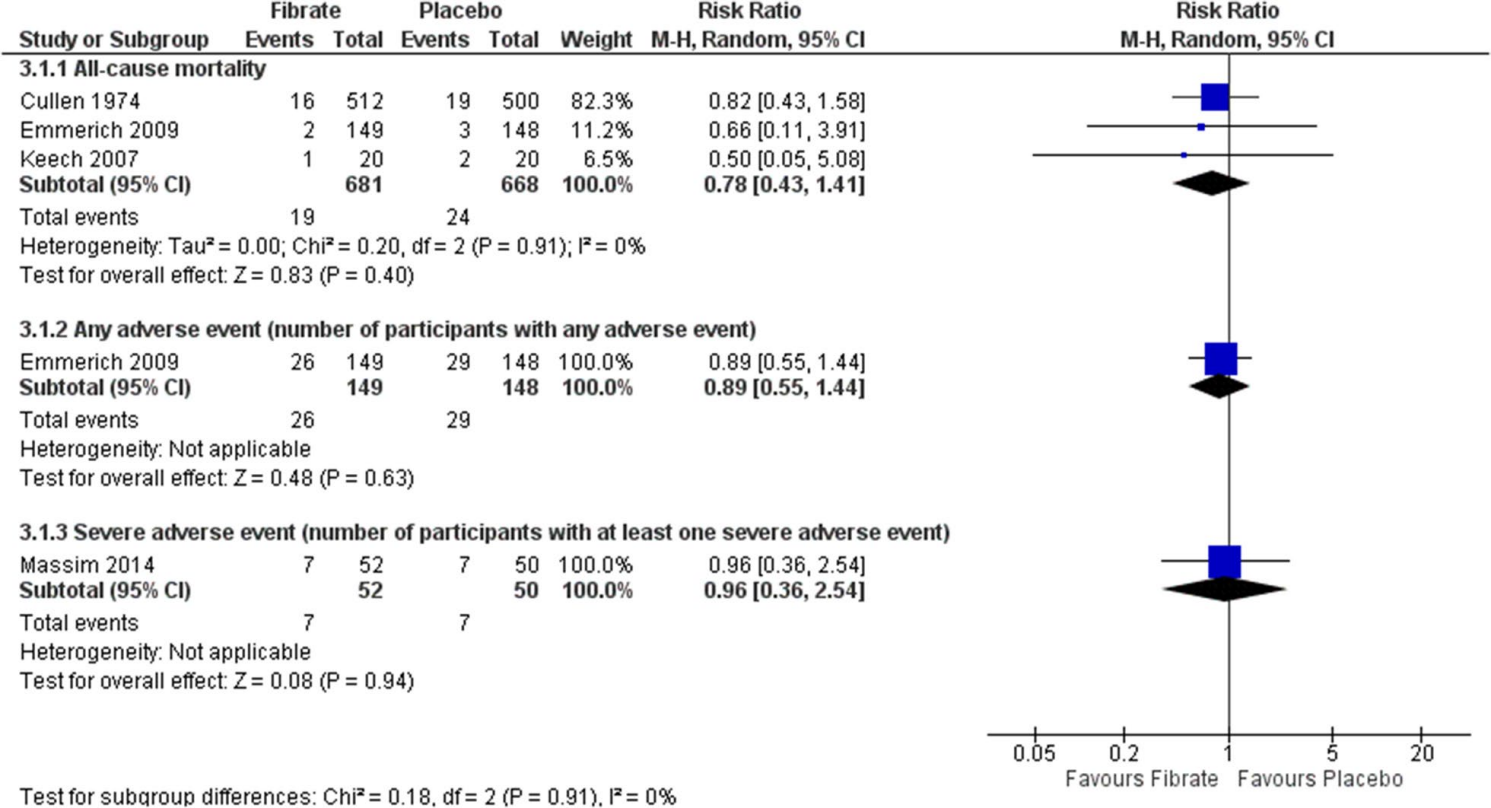
Comparison: fibrate versus placebo. Outcomes: adverse events

Four RCTs assessed this outcome [41, 42, 44]. No difference between interventions was found for mortality (RR 0.78, 95% CI 0.43 to 1.41; participants = 1349; studies = 3; I2 = 0%; low certainty of the evidence), rate of participants with any adverse event (RR 0.89, 95% CI 0.55 to 1.44; participants = 297; studies = 1; I2 = 0%; very low certainty of the evidence) and rate of participants with severe adverse event (RR 0.96, 95% CI 0.36 to 2.54; participants = 102; studies = 1; I^2^ = 0%; certainty of the evidence not assessed) (Fig. 8). The estimates around the adverse events were also imprecise or at low certainty of evidence, and future studies are likely to change the estimates.

None of included RCTs assessed quality of life.

### Fibrates/statin versus placebo/statin

One study assessed this comparison [40], with a 4-year follow-up. No difference between interventions was found for incidence of DR (ETDRS) (odds ratio [OR] 1.10; 95% CI 0.71 to 1.69; participants = 776; one study; very low certainty evidence). Also no difference was found for progression of DR assessed by (a) proportion of participants with retinopathy progression, two or more steps of ETDRS (OR 1.14; 95% CI 0.74 to 1.77; participants = 776; one study; low certainty of the evidence), (b) proportion of participants worsening macular edema assessed by ETDRS-DME (OR 1.08; 95% 0.60 to 1.94; participants = 1.570; one study; low certainty of the evidence), (c) proportion of participants worsening hard exudates (OR 1.14; 95% 0.60 to 2.16; participants = 1.521; one study; low certainty of the evidence). No difference was found for visual acuity (worsening of more than 15 letter, Logarithm of the Minimum Angle of Resolution, LogMAR) (Hazard Ratio [HR] 0.94; 95% CI 0.8 to 1.1; moderate certainty of the evidence) and progression for proliferative disease (participant referred to photocoagulation) (OR 0.51; 95% CI 0.22 to 1.22; participants = 1.583; one study; low certainty of the evidence). This RCT did not assessed quality of life neither reported adverse events. All of the outcomes assessment had a wide confidence interval, meaning that important reduction or increase in the outcomes cannot be ruled out based on the available data.

## Discussion

This systematic review found eight RCTs that fulfilled our eligibility criteria assessing the effects of fibrates (n = 4), statins (n = 3) and fibrate plus statins (n = 1) for therapy (n = 8) or prevention (n = 4) of DR. For statins, the quantitative and qualitative synthesis showed that we are uncertain about its effects and no conclusions could be draw due to poor methodological quality and imprecision raised up by the RCTs (incidence and progression of DR and visual acuity) nor by lack of measurement and/or reporting (quality of life, adverse events and progression to proliferative disease).

Fibrates seemed to be associated with a 45% risk reduction of macular edema incidence (ranging from 62 a 19%, but the confidence on this estimate is low, which means that is very likely that further studies can modify this estimate). The certainty of evidence for other outcomes was also very low or low, and we are uncertain regarding the effects of fibrates for DR (not considering quality of life that was not measured). Overall, the rate of adverse events seemed to be similar between fibrate and placebo, but again based on the width of the confidence intervals, an important increase of adverse events cannot be rule out.

The combination statin/fibrate did not seem to have benefit for visual acuity but is likely that further studies can modify this estimate since the current evidence is limited due to attrition bias and imprecision. Adverse events and quality of life were not measured or reported.

Some similar systematic reviews have been published on this topic [48, 49]. Das e cols considered RCTs, cohort, case–control, and cross-sectional studies to investigate the relation between blood lipid levels and diabetic macular edema, including the effects of lipid-lowering drugs for incidence and progression of DR. The electronic search was limited to two databases, with no hand or grey literature search and probably due to these flaws two relevant RCTs were not included [46, 47]. Furthermore, the efficacy analysis was restricted to incidence and progression of DR, based on ETDRS. Visual acuity, adverse events and quality of life were neglected outcomes. Shi and cols used non- recommended methods for quantitative synthesis, as pooling studies with different study designs (cohort and RCT) and using fixed-effects model as default. Concerns also exist around gathering studies with clinical diversity, as those did by the authors when assessing lipid- lowering drugs with different mechanisms of action. Finally, none of these systematic reviews evaluated the certainty around the body of the final evidence by GRADE approach. Similarly with our findings, the systematic reviews above found no benefit of lipid- lowering drugs for DR, although the methodological rigour of our review improves the confidence on these results.

This review was developed following the methodological rigor proposed by the Cochrane Handbook [15], it was prospectively recorded in the PROSPERO database and also was reported following PRISMA Statement [16]. We also did not identify any systematic review conducted to answer the same clinical question, that considered only RCTs and presented results separately for statins and fibrates. Our search was comprehensive and included electronic search, hand search, grey literature, and clinical trial register databases. In addition to assessing the risk of bias of primary studies, we summarized the certainty in the final body of evidence for each outcome using the GRADE approach.

However, this review has some weaknesses. Most of these limitations are related to the methodological quality of the included studies or to the way in which they were reported, rather than to the conduct of the review itself. The quality of the included studies was limited mainly due to uncertainty regarding the use of adequate methods to guarantee the allocation concealment, high risk of attrition bias (losses) and uncertainty regarding the selective reporting of outcomes, since only one RCT presented a prospectively registered protocol. The included RCTs were clinically heterogeneous regarding the unit of analysis (individual or eye), the outcomes and methods for measure them. The largest study included [40] was not planned to evaluate ophthalmologic outcomes. In addition, many studies have not revealed important characteristics of the population of interest as time to diabetes and retinopathy diagnosis. We tried to minimize this lack of information by contacting the authors directly for further information, however we did not get answers.

Eight studies identified in our search remained classified as ‘awaiting classification’—some because they presented results of proliferative and nonproliferative retinopathy combined [32, 33] and others because they were not available in an accessible format in the literature (abstract or full text). In both cases all the strategies for reclassification of the study (including contact of all the authors by e-mail and search in the journal website) were exhausted.

As implications for practice, this review suggests that fibrate appears to prevent the development of macular edema, but without benefits for visual acuity and progression to proliferative DR. Since there is an uncertainty regarding the risk of adverse events related to the use of fibrate for this purpose, its routine use in clinical practice for the prevention and treatment of DR cannot be recommended in the light of current evidence. For statins, the results of our review were more disappointing, and no reasonable conclusion could be drawn about its use in this population. Because of the lack of data to support clinical recommendations in the use of statins and/or fibrates, the glycemic control should remain the main tool for the management of DR.

As implications for future research, this review brings important considerations, such as the tools used to measure ophthalmologic outcomes, which are mostly subjective. Studies to identify and standardize the most clinically relevant outcomes and tools are critical, as those disseminated by the COMET (Core Outcome Measures in Effectiveness Trials) initiative [50]. The limited methodological quality of available RCTs also demands well-designed and -conducted RCTs to identify, under low uncertainty, the role of statins and/or fibrates for DR.

The findings of this review highlights the uncertainties surrounding the effects of statins and/or fibrates for diabetic retinopathy still remain after 45 years from the publication of the first RCT that proposed to evaluate this clinical question [41]. These results are important because identifying and publishing the gaps avoid publication bias that is fundamental to underpin changes in decision-making and to guide future research as suggested in the last paragraphs.

### Amendments from published protocol

In the published protocol, we planned to perform fixed-effect meta-analysis in the presence of low number of studies or low heterogeneity. After the study selection process, we expected that the clinical and methodological diversity of the studies would be important and we decided to perform only random-effects model meta-analysis. We highlighted that because of the overall certainty of the evidence, this decision did not affected in the results or conclusions of this systematic review.

## Conclusions

This systematic review found eight RCTs, with limited methodological quality, that assessed the effects of fibrates and/or statins for DR. Based on our findings, we are uncertain about the effects of statin for this purpose. Fibrates seemed to reduce the incidence of macular edema (low certainty evidence) without increase adverse events (low to very low certainty evidence). For the other outcomes, the data were not sufficient for any conclusion.

## Supplementary information

**Additional file 1.** Search strategies for each electronic database.

**Additional file 2.** Excluded studies and reason for exclusions.

**Additional file 3.** Awaiting classification studies.

**Additional file 4.** Justification for risk of bias judgments of included RCTs.

**Additional file 5.** Summary of findings table (statins compared to placebo for diabetic retinopathy).

**Additional file 6.** Summary of findings table (fibrates compared to placebo for diabetic retinopathy).

**Additional file 7.** Summary of findings table (Fibrate plus statin compared to statin alone for diabetic retinopathy).

## Data Availability

Not applicable.

## References

[1] Leasher JL, Bourne RRA, Flaxman SR, Jonas JB, Keeffe J, Naidoo K (2016). Global estimates on the number of people blind or visually impaired by diabetic retinopathy: a meta-analysis from 1990 to 2010. Diabetes Care.

[2] Centers for Disease Control and Prevention. National Diabetes Statistics Report, 2017. https://www.cdc.gov/diabetes/data/statistics/statistics-report.html. Accessed 11 Jun 2019.

[3] Fong DS, Aiello LP, Ferris FL, Klein R (2004). Diabetic retinopathy. Diabetes Care..

[4] Dahl-Jørgensen K, Brinchmann-Hansen O, Hanssen KF, Sandvik L, Aagenaes O (1985). Rapid tightening of blood glucose control leads to transient deterioration of retinopathy in insulin dependent diabetes mellitus: the Oslo study. Br Med J.

[5] Kroc Collaborative Study Group (1984). Blood glucose control and the evolution of diabetic retinopathy and albuminuria. A preliminary multicenter trial. N Engl J Med..

[6] Evans JR, Michelessi M, Virgili G (2014). Laser photocoagulation for proliferative diabetic retinopathy. Cochrane Database Syst Rev..

[7] Jorge EC, Jorge EN, Botelho M, Farat JG, Virgili G, Dib R (2018). Monotherapy laser photocoagulation for diabetic macular oedema. Cochrane Database Syst Rev..

[8] Martinez-Zapata MJ, Martí-Carvajal AJ, Solà I, Pijoán JI, Buil-Calvo JA, Cordero JA (2014). Anti-vascular endothelial growth factor for proliferative diabetic retinopathy. Cochrane Database Syst Rev..

[9] Smith JM, Steel DH (2015). Anti-vascular endothelial growth factor for prevention of postoperative vitreous cavity haemorrhage after vitrectomy for proliferative diabetic retinopathy. Cochrane Database Syst Rev..

[10] Mehta H, Hennings C, Gillies MC, Nguyen V, Campain A, Fraser-Bell S (2018). Anti-vascular endothelial growth factor combined with intravitreal steroids for diabetic macular oedema. Cochrane Database Syst Rev..

[11] Grover D, Li TJ, Chong CC (2008). Intravitreal steroids for macular edema in diabetes. Cochrane Database Syst Rev..

[12] Kawasaki R, Konta T, Nishida K (2018). Lipid-lowering medication is associated with decreased risk of diabetic retinopathy and the need for treatment in patients with type 2 diabetes: a real-world observational analysis of a health claims database. Diabetes Obes Metab.

[13] Klein BE, Moss SE, Klein R, Surawicz TS (1991). The Wisconsin Epidemiologic Study of Diabetic Retinopathy. XIII. Relationship of serum cholesterol to retinopathy and hard exudate. Ophthalmology..

[14] Chew EY, Klein ML, Ferris FL, Remaley NA, Murphy RP, Chantry K (1996). Association of elevated serum lipid levels with retinal hard exudate in diabetic retinopathy Early Treatment Diabetic Retinopathy Study (ETDRS) Report 22. Arch Ophthalmol..

[15] Higgins JPT, Green S. Cochrane Handbook for Systematic Reviews of Interventions Version 5.1.0. The Cochrane Collaboration; 2011http://handbook.cochrane.org/. Accessed Mar 2011.

[16] Liberati A, Altman DG, Tetzlaff J, Mulrow C, Gøtzsche PC, Ioannidis JP (2009). The PRISMA statement for reporting systematic reviews and meta-analyses of studies that evaluate health care interventions: explanation and elaboration. J Clin Epidemiol.

[17] Mozetic V, Freitas CG, Riera R (2017). Statins and fibrates for diabetic retinopathy: protocol for a systematic review. JMIR Res Protoc..

[18] Early Treatment Diabetic Retinopathy Study Research Group (1991). Grading diabetic retinopathy from stereoscopic color fundus photographs—an extension of the modified Airlie House classification. ETDRS report number 10. Ophthalmology..

[19] Bailey IL, Lovie JE (1976). New design principles for visual acuity letter charts. Am J Optom Physiol Opt..

[20] Bailey IL, Lovie-Kitchin JE (2013). Visual acuity testing From the laboratory to the clinic. Vision Res..

[21] Ouzzani M, Hammady H, Fedorowicz Z, Elmagarmid A (2016). Rayyan—a web and mobile app for systematic reviews. Syst Rev..

[22] Review Manager (RevMan) [Software]. Version 5.3. Copenhagen: The Nordic Cochrane Centre, The Cochrane Collaboration, 2014.

[23] Atkins D, Best D, Briss PA, Eccles M, Falck-Ytter Y, Flottorp S (2004). Grading quality of evidence and strength of recommendations. BMJ.

[24] GRADEpro GDT: GRADEpro Guideline Development Tool [Software]. McMaster University, 2015 (developed by Evidence Prime, Inc.). Available from gradepro.org.

[25] Colhoun HM, Betteridge DJ, Durrington PN, Hitman GA, Neil HA, Livingstone SJ (2004). Primary prevention of cardiovascular disease with atorvastatin in type 2 diabetes in the Collaborative Atorvastatin Diabetes Study (CARDS): multicentre randomised placebo-controlled trial. Lancet.

[26] Cullen JF, Ireland JT, Oliver MF (1964). A controlled trial of Atromid therapy in exudative diabetic retinopathy. Trans Ophthalmol Soc U K..

[27] Duncan LJ, Cullen JF, Ireland JT, Nolan J, Clarke BF, Oliver MF (1968). A three-year trial of atromid therapy in exudative diabetic retinopathy. Diabetes.

[28] Grigoryeva N, Shklyarov E, Shadrichev F, Kryaneva O. Fenofibrate effect on diabetic retinopathy. In: 21st Meeting of the European Association for the Study of Diabetes. Gdańsk Poland. 2011.

[29] Ilyina Y, Bezditko P, Mohamed AS, Zavoloka O, Zubkova D (2016). Statins and fibrates as the treatment of nonproliferative diabetic retinopathy in type 2 diabetes mellitus. Spektrum der Augenheilkunde..

[30] Ueshima K, Itoh H, Kanazawa N, Komuro I, Nagai R, Takeuchi M (2016). Rationale and design of the standard versus intensive statin therapy for hypercholesterolemic patients with diabetic retinopathy (EMPATHY) study: a randomized controlled trial. J Atheroscler Thromb..

[31] Vannas S, Esilä R, Tuovinen E (1968). Observations on the effect of ethyl a-p chlorophenoxyisobutyrate (CPIB) therapy on serum lipid levels and on diabetic and some other retinopathies. Acta Ophthalmol (Copenh)..

[32] Harrold BP, Marmion VJ, Gough KR (1969). A double-blind controlled trial of clofibrate in the treatment of diabetic retinopathy. Diabetes.

[33] Fried LF, Forrest KY, Ellis D, Chang Y, Silvers N, Orchard TJ (2001). Lipid modulation in insulin-dependent diabetes mellitus: effect on microvascular outcomes. J Diabetes Complications.

[34] Raic N, Panajatovic N, Skoflic A (1973). Atromidin in the treatment of diabetic retinopathy (Serbocroatian). Diabetologia Croatica..

[35] Rjasanowski J, Bruns W (1979). Clofibrate in the treatment of diabetic retinopathy. Vestn oftalmol.

[36] Kliachko VR, Tirkina TN, Aleksandrova LM, Mazovetskiĭ AG (1973). Use of atromide-S in the treatment of diabetic retinopathy. Sov Med..

[37] Malcolm J, Lockwood PW, Comhaire E, Malcolm-Thomas B (1973). Clofibrate in the treatment of exudative diabetic retinopathy. Brux Med..

[38] Margolis MG, Zefirova GS, Tomenko LP (1972). Shmushkovich VI [Atromid and its analogs in the complex treatment of patients with diabetes mellitus and retinopathy]. Vestn oftalmol.

[39] Tiholov K, Lukov L (1974). Treatment of diabetic retinopathy with calcium dobesilate, clofibrate and biguanide derivatives (Bulgarian). Ophthalmologia..

[40] Chew EY, Davis MD, Danis RP, Lovato JF, Perdue LH, Greven C (2014). The effects of medical management on the progression of diabetic retinopathy in persons with type 2 diabetes: the Action to Control Cardiovascular Risk in Diabetes (ACCORD) Eye Study. Ophthalmology.

[41] Cullen JF, Town SM, Campbell CJ (1974). Double-blind trial of Atromid-S in exudative diabetic retinopathy. Trans Ophthalmol Soc UK..

[42] Emmerich KH, Poritis N, Stelmane I, Klindzane M, Erbler H, Goldsteine J (2009). Efficacy and safety of etofibrate in patients with non-proliferative diabetic retinopathy. Klin Monbl Augenheilkd..

[43] Gupta A, Gupta V, Thapar S, Bhansali A (2004). Lipid-lowering drug atorvastatin as an adjunct in the management of diabetic macular edema. Am J Ophthalmol.

[44] Keech AC, Mitchell P, Summanen PA, O’Day J, Davis TM, Moffitt MS (2007). Effect of fenofibrate on the need for laser treatment for diabetic retinopathy (FIELD study): a randomised controlled trial. Lancet.

[45] Massin P, Peto T, Ansquer JC, Aubonnet P (2014). MacuFEN Study Investigators Effects of fenofibric acid on diabetic macular edema: the MacuFen study. Ophthalmic Epidemiol..

[46] Narang S, Sood S, Kaur B, Singh R, Mallik A, Kaur J (2012). Atorvastatin in clinically-significant macular edema in diabetics with a normal lipid profile. Nepal J Ophthalmol..

[47] Sen K, Misra A, Kumar A, Pandey RM (2002). Simvastatin retards progression of retinopathy in diabetic patients with hypercholesterolemia. Diabetes Res Clin Pract.

[48] Das R, Kerr R, Chakravarthy U, Hogg RE (2015). Dyslipidemia and diabetic macular edema: a systematic review and meta-analysis. Ophthalmology.

[49] Shi R, Zhao L, Wang F, Liu F, Chen Z, Li R (2018). Effects of lipid-lowering agents on diabetic retinopathy: a meta-analysis and systematic review. Int J Ophthalmol..

[50] Williamson PR, Altman DG, Bagley H, Barnes KL, Blazeby JM, Brookes ST (2017). The COMET Handbook: version 1.0. Trials..

