# Supplementary material for: Statins and/or fibrates for diabetic retinopathy: a systematic review and meta-analysis
Source: Diabetol Metab Syndr. 2019 Nov 8;11:92. doi: 10.1186/s13098-019-0488-9 (PMC6839185; doi:10.1186/s13098-019-0488-9)
Supplement: Supplementary file 2 — Additional file 2. Excluded studies and reason for exclusions. [file 13098_2019_488_MOESM2_ESM.docx]

**Additional file 2.** Excluded studies and reason for exclusions.

| **Study** | **Reason** |
| --- | --- |
| Colhoun 2004 | None ophthalmologic outcomes planned. |
| Cullen 1964 | Quasi-randomized study. |
| Duncan 1968 | Quasi-randomized study. |
| Grigoryeva 2011 | No randomized comparative study. |
| Iliyna 2016 | Probably not randomized. We contacted the authors for further information and an unclear answer about the method for randomization was provided. |
| Ueshima 2016 | None ophthalmologic outcomes planned. |
| Vannas 1968 | Uncontrolled experimental study. |
