# Supplementary material for: Statins and/or fibrates for diabetic retinopathy: a systematic review and meta-analysis
Source: Diabetol Metab Syndr. 2019 Nov 8;11:92. doi: 10.1186/s13098-019-0488-9 (PMC6839185; doi:10.1186/s13098-019-0488-9)
Supplement: Supplementary file 3 — Additional file 3. Awaiting classification studies. [file 13098_2019_488_MOESM3_ESM.docx]

**Additional file 3.** Awaiting classification studies

| **Study** | **Reason** |
| --- | --- |
| Harrold 1969 | Results for proliferative and nonproliferative retinopathy were presented together. We did not have contact with authors. |
| Fried 2001 | Results for proliferative and nonproliferative retinopathy were presented together. We sent two messages (2016, 28th December 2018) for contact authors requesting separated data and received no reply so far. |
| Raic 1973 | Study not found even after trying contact with authors by e-mail and searching at the journal website. |
| Rjasanowski 1979 | Study not found even after trying contact with authors by e-mail and searching at the journal website. |
| [Kliachko](https://rayyan.qcri.org/authors/4920942) 1973 | Study not found even after trying contact with authors by e-mail and searching at the journal website. |
| [Malcolm](https://rayyan.qcri.org/authors/3844239) 1973 | Study not found even after trying contact with authors by e-mail and searching at the journal website. |
| [Margolis](https://rayyan.qcri.org/authors/3311109) 1972 | Study not found even after trying contact with authors by e-mail and searching at the journal website. |
| [Tiholov](https://rayyan.qcri.org/authors/13894277) 1974 | Study not found even after trying contact with authors by e-mail and searching at the journal website. |
