# Supplementary material for: Statins and/or fibrates for diabetic retinopathy: a systematic review and meta-analysis
Source: Diabetol Metab Syndr. 2019 Nov 8;11:92. doi: 10.1186/s13098-019-0488-9 (PMC6839185; doi:10.1186/s13098-019-0488-9)
Supplement: Supplementary file 4 — Additional file 4. Justification for risk of bias judgments of included RCTs. [file 13098_2019_488_MOESM4_ESM.docx]

**Additional file 4.** Justification for risk of bias judgments of included RCTs.

| **Criteria/ Study** | **Chew**  **2014**  **(ACCORD Eye)** | **Cullen 1974** | **Emmerich 2009** | **Gupta**  **2004** | **Keech 2007 (FIELD Sudy)** | **Massim 2014 (MacuFen Study)** | **Narang 2012** | **Sen 2002** |
| --- | --- | --- | --- | --- | --- | --- | --- | --- |
| Random sequence generation (selection bias) | **Low**  **Quote:** *‘An internet-based,*  *web browser randomization”*  **Comment:** described and adequate. | **Low**  **Quote:** *‘Utilizing a system of random selection’* **Comment:** described and adequate. | **Unclear**  **Quote:** ‘*Studiendesign Es handelte sich um eine randomisierte, plazebokontrollierte Doppelblindstudie in 2 Zentren in Lettland.’*  **Comment:** it does not specify the random method. | **Low**  **Quote:** *‘the patients were ranodomized into two groups, and B, by means of tippet random tables’.* **Comment:** described and adequate. | **Low**  **Quote:** ‘*randomization was carried out using a dynamic allocation method with stratification for important prognostic factors, including age, sex, prior MI, lipid levels and urinary albumin excretion’*  **Comment:** described and adequate**.** | **Unclear**  **Quote:** ‘*Male and female subjects with type 2 diabetes mellitus and DME were included in a multicenter (31 centers in Europe), randomized, prospective, double-blind, placebo-controlled study.’*  **Comment**: it does not specify the random method. | **Low**  **Quote:** *‘The patients were randomized into two groups of 15 patients each using random dot tables’* **Comment:** described and adequate. | **Low**  **Quote:** *‘…an independent person randomized the patients two weeks later using a random number table’*  **Comment:** described and adequate. |
| Allocation concealment (selection bias) | **Low**  **Quote:** *‘An internet-based,*  *web browser randomization’*  **Comment:** described and adequate. | **Unclear**  **Comment:** insufficient information for judgment. | **Unclear**  **Comment:** insufficient information for judgment. | **Unclear**  **Comment:** insufficient information for judgment. | **Low**  **Quote:** *‘randomisation was done by central computer, using a dynamic allocation method with stratification for important prognostic factors, including age, sex, previous myocardial infarction, lipid levels, and urinary albumin concentration.´*  **Comment:**  described and adequate. | **Unclear**  **Comment:** insufficient information for judgment. | **Unclear**  **Comment:** insufficient information for judgment. | **Unclear**  **Comment:** insufficient information for judgment. |
| Blinding of participants and personnel (performance bias) | **Low**  **Quote:** *‘The lipid component of ACCORD is a fully masked, randomized trial of 5,800 participants’.*  **Comment:** described and adequate. | **Low**  **Quote:** *‘…an equal number of identical placebo’*.  **Comment:** described and adequate. | **Low**  **Quote:** ‘*Die Tabletten, Etofibrat und Plazebo, waren hinsichtlich Farbe, Form, Geruch und Geschmack identisch.*’  **Comment:** described and adequate. | **Low**  (retinopathy progression)  **Unclear**  (visual acuity)  **Quote:** ‘*…member of group B did not received any lipid-lowering therapy’*.  **Comment:** it is not clear if placebo was used. Visual acuity assessment, but not a and retinopathy progression, could be influenced by this fact | **Low**  **Quote:** ‘*9795 patients were eligible for inclusion, and were randomly assigned to receive micronised fenofi brate 200 mg once daily (Laboratoires Fournier, Dijon, France) or matching placebo*.’  **Comment:** described and adequate. | **Low**  **Quote:** *‘All capsules were identical in appearance, shape, smell and taste, and packaged in the proper proportion to assure desired dosages and maintenance of the blind during the study*.’  **Comment:** described and adequate. | **Unclear**  **Comment:** insufficient information for judgment | **Low**  **Quote:** ‘*both tablets were identical in appearance*’  **Comment:** described and adequate. |
| Blinding of outcome assessment (detection bias) | **Low**  **Quote:** *‘The lipid component of ACCORD is a fully masked, randomized trial of 5,800 participants’.*  **Comment:** described and adequate | **Low**  **Quote:** ‘…*an impartial observer who had not seen the patients initially evaluated the clinical progress…’*  **Comment:** described and adequate | **Low**  **Quote:** ‘*Die Tabletten, Etofibrat und Plazebo, waren hinsichtlich Farbe, Form, Geruch und Geschmack identisch.*’  **Comment:** described and adequate | **Low**  **Quote:** ‘*Initially done by two graders who were masked to the treatment protocol’*  **Comment:** described and adequate | **Low**  **Quote**: ‘*Grading of retinopathy and macular oedema was done by the study ophthalmologists (PM, PAS), or a trained photographic grader (MSM), who were masked to treatment allocation.*’  **Comment:** described and adequate | **Unclear**  **Comment:** insufficient information for judgment | **Unclear**  **Comment:** insufficient information for judgment | **Low**  **Quote:** *‘At the end of the study, the randomization code was opened in the presence of all the members of the team.’*  **Comment:** described and adequate |
| Incomplete outcome data addressed (attrition bias) | **High**  **Quote:** *‘From October 2003 to February 2006, 3472 eligible participants were enrolled in the ACCORD Eye Study. Of these 2856 (85% of survivors) returned for the second eye examination and fundus photographs.’*  **Comment:** there was more than 10% of losses | **High**  **Quote:** ‘*Of the forty patients, 32 completed the trial’*  **Comment:** there was 20% of losses | **High**  **Comment:** loss of 48/148 in intervention group and 30/148 in control group. High rate of losses with an imbalance between groups. | **Low**  **Comment:** there was no loss | **High**  **Quote:** ‘*Of 1012 participants, 850 were assessed at the end of the study’.*  **Comment:** there was 16% of losses | **Low**  **Quote:** *‘The efficacy analysis was performed on the completer set, with 52 and 50 subjects in the fenofibric acid and placebo groups’*  **Comment:** there was 7.3% of losses | **Low**  **Comment:** there was no loss | **High**  **Quote:** ‘*As per the design and protocol of the study, four patients in the placebo group were removed from the study as their VA had worsened and eyes showed changes of*  *CSME. The removal was done at the completion*  *of 90 days of follow-up, and data were included in*  *the final analysis.’*  **Comment:** There were 4/25 losses in intervention group and no losses in control group (imbalance between groups) |
| Selective reporting (reporting bias) | **Unclear**  **Comment:** study retrospectively registered in clinical trials database.  NCT00542178 | **Unclear**  **Comment:** no protocol available | **Unclear**  **Comment:** no protocol available. | **Unclear**  **Comment:** no protocol available. | **Unclear**  **Comment:** study retrospectively registered in clinical trials database.  ISRCTN64783481 | **Low**  **Comment:**  the reporting is consistent with the protocol.  NCT00683176 | **Unclear**  **Comment:** no protocol available. | **Unclear**  **Comment:** no protocol available. |
| Other bias | **Low**  **Comment:** no other source of bias was found. | **Low**  **Comment:** no other source of bias was found. | **Low**  **Comment:** no other source of bias was found. | **Low**  **Comment:** no other source of bias was found. | **Low**  **Comment:** no other source of bias was found. | **Low**  **Comment:** no other source of bias was found. | **Low**  **Comment:** no other source of bias was found. | **Low**  **Comment:** no other source of bias was found. |
